# Prediagnosis Leisure-Time Physical Activity and Lung Cancer Survival: A Pooled Analysis of 11 Cohorts

**DOI:** 10.1093/jncics/pkac009

**Published:** 2022-02-14

**Authors:** Jae Jeong Yang, Danxia Yu, Emily White, Dong Hoon Lee, William Blot, Kim Robien, Rashmi Sinha, Yikyung Park, Yumie Takata, Yu-Tang Gao, Karl Smith-Byrne, Evelyn M Monninkhof, Rudolf Kaaks, Arnulf Langhammer, Kristin Benjaminsen Borch, Laila Al-Shaar, Qing Lan, Elin Pettersen Sørgjerd, Xuehong Zhang, Clair Zhu, María Dolores Chirlaque, Gianluca Severi, Kim Overvad, Carlotta Sacerdote, Dagfinn Aune, Mattias Johansson, Stephanie A Smith-Warner, Wei Zheng, Xiao-Ou Shu

**Affiliations:** 1 Vanderbilt Epidemiology Center, Division of Epidemiology, Department of Medicine, Vanderbilt-Ingram Cancer Center, Vanderbilt University Medical Center, Nashville, TN, USA; 2 Cancer Prevention Program, Fred Hutchinson Cancer Research Center, Seattle, WA, USA; 3 Departments of Nutrition and Epidemiology, Harvard T.H. Chan School of Public Health, Boston, MA, USA; 4 Department of Exercise and Nutrition Sciences, Milken Institute School of Public Health, George Washington University, Washington, DC, USA; 5 Division of Epidemiology & Genetics, National Cancer Institute, Bethesda, MD, USA; 6 Division of Public Health Sciences, Department of Surgery, Washington University School of Medicine, St. Louis, MO, USA; 7 Program of Nutrition, School of Biological and Population Health, College of Public Health and Human Sciences, Oregon State University, Corvallis, OR, USA; 8 Department of Epidemiology, Shanghai Cancer Institute, Renji Hospital, Shanghai Jiaotong University School of Medicine, Shanghai, China; 9 Genetic Epidemiology Group, International Agency for Research on Cancer, Lyons, France; 10 Julius Center for Health Sciences and Primary Care, University Medical Center, Utrecht University, Utrecht, the Netherlands; 11 Department of Cancer Epidemiology, German Cancer Research Center (DKFZ), Heidelberg, Germany; 12 Translational Lung Research Center (TLRC), Member of the German Center for Lung Research (DZL), Heidelberg, Germany; 13 HUNT Research Centre, Department of Public Health and Nursing, NTNU, Norwegian University of Science and Technology, Levanger, Norway; 14 Levanger Hospital, Nord-Trøndelag Hospital Trust, Levanger, Norway; 15 Department of Community Medicine, UiT—the Arctic University of Norway, Tromsø, Norway; 16 Department of Public Health Sciences, Penn State College of Medicine, Hershey, PA, USA; 17 Department of Public Health and General Practice, Norwegian University of Science and Technology, Trondheim, Norway; 18 Channing Division of Network Medicine, Department of Medicine, Brigham and Women’s Hospital, Harvard Medical School, Boston, MA, USA; 19 Department of Epidemiology, Murcia Regional Health Council IMIBArrixaca, Ronda de Levante, Murcia, Spain; 20 Department of Health and Social Sciences, University of Murcia Campus de Espinardo, Murcia, Spain; 21 CIBER Epidemiología y Salud Pública (CIBERESP), Calle de Melchor Fernández Almagro, Madrid, Spain; 22 University Paris-Saclay, UVSQ, Inserm, Gustave Roussy, “Exposome and Heredity” Team, CESP UMR1018, Villejuif, France; 23 Department of Statistics, Computer Science and Applications “G. Parenti” (DISIA), University of Florence, Italy; 24 Department of Public Health, Aarhus University, Aarhus, Denmark; 25 Unit of Cancer Epidemiology, Città della Salute e della Scienza University-Hospital, Turin, Italy; 26 Department of Epidemiology and Biostatistics, School of Public Health, Imperial College London, London, UK; 27 Department of Nutrition, Bjørknes University College, Oslo, Norway; 28 Department of Endocrinology, Morbid Obesity and Preventive Medicine, Oslo University Hospital, Oslo, Norway

## Abstract

**Background:**

Little is known about the association between physical activity before cancer diagnosis and survival among lung cancer patients. In this pooled analysis of 11 prospective cohorts, we investigated associations of prediagnosis leisure-time physical activity (LTPA) with all-cause and lung cancer–specific mortality among incident lung cancer patients.

**Methods:**

Using self-reported data on regular engagement in exercise and sports activities collected at study enrollment, we assessed metabolic equivalent hours (MET-h) of prediagnosis LTPA per week. According to the Physical Activity Guidelines for Americans, prediagnosis LTPA was classified into inactivity, less than 8.3 and at least 8.3 MET-h per week (the minimum recommended range). Cox regression was used to estimate hazard ratios (HRs) and 95% confidence interval (CIs) for all-cause and lung cancer–specific mortality after adjustment for major prognostic factors and lifetime smoking history.

**Results:**

Of 20 494 incident lung cancer patients, 16 864 died, including 13 596 deaths from lung cancer (overall 5-year relative survival rate = 20.9%, 95% CI = 20.3% to 21.5%). Compared with inactivity, prediagnosis LTPA of more than 8.3 MET-h per week was associated with a lower hazard of all-cause mortality (multivariable-adjusted HR = 0.93, 95% CI = 0.88 to 0.99), but not with lung cancer–specific mortality (multivariable-adjusted HR = 0.99, 95% CI = 0.95 to 1.04), among the overall population. Additive interaction was found by tumor stage (*P*_interaction_ = .008 for all-cause mortality and .003 for lung cancer–specific mortality). When restricted to localized cancer, prediagnosis LTPA of at least 8.3 MET-h per week linked to 20% lower mortality: multivariable-adjusted HRs were 0.80 (95% CI = 0.67 to 0.97) for all-cause mortality and 0.80 (95% CI = 0.65 to 0.99) for lung cancer–specific mortality.

**Conclusions:**

Regular participation in LTPA that met or exceeded the minimum Physical Activity Guidelines was associated with reduced hazards of mortality among lung cancer patients, especially those with early stage cancer.

Lung cancer is the most common cancer in the world and accounts for approximately 2.09 million new cases and 1.76 million deaths in 2018 (1). Despite the recent advances in lung cancer screening and treatments, more than half of newly diagnosed patients die within a year of diagnosis; the overall 5-year survival rate of lung cancer remains under 20% worldwide (2,3). To reduce the global burdens of lung cancer, it is crucial to identify potential risk and prognostic factors apart from smoking cessation.

Physical activity (PA) has attracted great attention in cancer research because of its benefits in reducing inflammation, regulating hormones (eg, insulin), and improving immune function and energy balance (4-6). Epidemiological evidence to date supports a link of PA to cancer prevention and survival (7-11). The Physical Activity Guidelines Advisory Committee and the American College of Sports Medicine Roundtable have recently concluded that PA prevents at least 7 cancers (eg, breast and colon) and might confer survival benefits to patients with breast, colon, and prostate cancers (8,9,11,12). Yet, evidence on PA and lung cancer remains moderate or limited, especially for survival outcomes (ie, all-cause and lung cancer–specific mortality) (8,9,11,12). Although a recent meta-analysis and some prospective studies have shown a statistically significant reduction in lung cancer mortality attributed to prediagnosis PA (7,13-15), most of these studies had a small sample size. They are also limited by residual confounding because of smoking and lack of consideration of major prognostic factors and are unable to address subgroup variations. Large-scale, population-based prospective investigations that overcome previous limitations are needed to fill a research gap and provide convincing evidence.

To this end, this pooled analysis of 11 cohorts from the United States (US), Europe, and Asia aims to investigate associations of prediagnosis leisure-time PA (LTPA) with all-cause and lung cancer–specific mortality among more than 20 000 incident primary lung cancer patients. Given the substantial difference in survivorship across lung cancer stage (2,3), analyses were conducted in the overall study population and subgroups defined by tumor stage. Furthermore, we assessed effect modification by established prognostic factors (ie, stage, histology, and grade), time interval from LTPA assessment to cancer diagnosis (given possible measurement errors or biologically relevant time windows), lifetime smoking history, and other risk factors.

## Methods

### Study Populations

We harmonized de-identified, individual participant data from 11 cohorts (16,17), including 7 US cohorts (National Institute of Health–American Association of Retired Persons Diet and Health Study; Health Professionals Follow-up Study; Nurses’ Health Study; Iowa Women’s Health Study; Prostate, Lung, Colorectal, and Ovarian Cancer Screening Trial; Southern Community Cohort Study; and VITamins And Lifestyle Study), 2 European cohorts (European Prospective Investigation into Cancer and Nutrition and Trøndelag Health Study), and 2 Asian cohorts (Shanghai Men’s Health Study and Shanghai Women’s Health Study). All studies were approved by the institutional review boards and ethics committees of the hosting institutes.

Among 1 588 378 initial participants, we identified 22 762 incident lung cancer patients diagnosed after study enrollment. Of those, we excluded individuals who had no data on LTPA (n = 2031), smoking history (n = 183), and survival time (n = 45). Cancer in situ was also excluded (n = 9), thus leaving 20 494 patients. Characteristics of each participating cohort and our analytic sample are summarized in Table 1.

**Table 1.** Participating cohorts included in the pooled analysis of prediagnosis leisure-time physical activity and lung cancer survival

Cohorts	No. of cases^a^	No. of deaths^b^	Year of diagnosis	Median time intervals (IQR), y^c^	Mean age at diagnosis (SD), y	Meet the guideline, %^d^	Median LTPA (IQR), MET-h/week	Women, %	Smokers, %^e^	NSCC, %	5-year survival Rate (95% CI), %
USA											
AARP	9684	8407 (6615)	1995-2007	5.5 (2.9-8.1)	68.8 (5.5)	39.2	4.5 (0.3-10.5)	36.9	93.1	83.5	20.1 (19.3 to 20.9)
HPFS	983	885 (812)	1986-2009	12.4 (6.8-17.4)	72.9 (8.8)	51.5	8.8 (2.8-22.1)	0.0	88.8	85.3	22.5 (20.0 to 25.2)
NHS	1542	1257 (1171)	1986-2009	13.9 (8.6-18.9)	69.4 (7.7)	40.4	5.2 (2.0-15.2)	100.0	90.8	83.9	27.3 (25.0 to 29.6)
IWHS	1017	914 (741)	1986-2012	12.8 (7.0-18.8)	73.9 (7.5)	41.9	8.2 (0.8-24.8)	100.0	82.3	79.7	16.1 (13.9 to 18.4)
PLCO	666	263 (237)	1995-2010	7.5 (4.8-9.3)	71.2 (6.1)	34.1	4.5 (0.7-12.6)	49.9	89.6	90.0	53.8 (49.5 to 57.9)
SCCS	815	585 (481)	2002-2013	3.6 (1.9-5.7)	60.2 (8.7)	17.2	0.0 (0.0-0.1)	46.3	94.8	84.6	18.9 (16.1 to 21.8)
VITAL	1029	764 (658)	2000-2012	5.2 (0.2-11.4)	71.1 (7.1)	32.7	3.3 (0.2-11.4)	45.7	92.5	86.1	21.1 (18.6 to 23.8)
Europe											
EPIC	2540	2081 (1666)	1992-2009	7.1 (4.0-9.7)	64.9 (8.0)	81.5	22.5 (12.0-42.0)	45.4	90.0	81.0	15.0 (13.6 to 16.5)
HUNT	474	432 (NA)	1995-2011	8.4 (4.8-11.6)	68.9 (10.5)	25.5	3.4 (2.3-8.6)	40.7	94.1	80.0	10.4 (7.85 to 13.4)
Asia											
SMHS	918	706 (684)	2002-2015	5.8 (3.2-8.3)	67.0 (9.5)	30.3	0.0 (0.0-11.2)	0.0	85.8	87.1	16.5 (14.1 to 19.1)
SWHS	826	570 (531)	1997-2015	10.2 (6.2-13.5)	67.2 (9.0)	26.4	0.0 (0.0-8.4)	100.0	8.0	99.0	26.0 (22.9 to 29.2)
Total	20 494	16 864 (13 596)	1986-2015	7.1 (3.5-9.5)	68.5 (7.6)	42.6	4.5 (0.7-15.0)	46.3	88.0	84.1	20.9 (20.3 to 21.5)

a Including primary lung cancer patients who were eligible for the current pooled analysis. AARP = National Institute of Health–American Association of Retired Persons Diet and Health Study; CI = confidence interval; EPIC = European Prospective Investigation into Cancer & Nutrition; HPFS = Health Professionals Follow-up Study; HUNT = Trøndelag Health Study; IQR = interquartile range; IWHS = Iowa Women’s Health Study; LTPA = leisure-time physical activity; MET-h/week = metabolic equivalent hours per week; NHS = Nurses’ Health Study; NSCC = non-small cell carcinoma; PLCO = Prostate, Lung, Colorectal and Ovarian Cancer Screening Trial; SCCS = Southern Community Cohort Study; VITAL = VITamins And Lifestyle Study; SMHS = Shanghai Men’s Health Study; SWHS = Shanghai Women’s Health Study.

b Number of deaths from all causes (deaths from lung cancer).

c Years from leisure-time physical activity assessment to lung cancer diagnosis.

d Percentage of adherence to the minimum recommended range of the Physical Activity Guidelines at least 500 MET-min (8.3 MET-h) per week.

e Including current and former smokers.

### Assessment and Parameterization of LTPA Exposure

This study used LTPA data only, not incorporating other domains of PA. Prediagnosis LTPA was assessed at baseline using validated cohort-specific questionnaires asking about regular engagement in exercise and sports activities (18-26). Details of LTPA assessment in each cohort, including original questions, intensity, and exposure windows, are shown in Supplementary Table 1 (available online). The level of LTPA was quantified in metabolic equivalent hours per week (MET-h/week), using the Compendium of Physical Activities (27). Based on the Physical Activity Guidelines for Americans (12,28,29), prediagnosis LTPA was classified into inactive, low active (>0 to <8.3 MET-h/week), moderately active (the recommended level for health benefits: 8.3-16.0 MET-h/week, equivalent to 150-300 minutes of moderate or 75-150 minutes of vigorous intensity activity per week), and highly active (>16.0 MET-h/week). Because of the limited number of study participants in the highly active group, this group was finally combined with the moderately active group, referred to as met or exceeded the minimum recommendation (≥8.3 MET-h/week).

### Assessment of Outcome

Each cohort has followed-up cancer incidence and mortality via linkages to national and regional registries, follow-up surveys, medical record reviews, or a combination of these methods. Incident lung cancer patients were ascertained using the *International Classification of Diseases* 9th or 10th revision (162 and C34, respectively). All patients were subclassified by histological type (adenocarcinoma, squamous cell carcinoma, other non-small cell carcinoma, small cell carcinoma, or unspecified or unknown), stage (localized [stage I/II], regional [stage III], distant [stage IV], and unknown), and grade (well, moderately, and poorly differentiated; undifferentiated; and unknown). For deceased ones, we obtained information on the underlying cause and date of death. Survival time was calculated as total years from the date of lung cancer diagnosis to the date of death, loss-to-follow-up, or end of follow-up, whichever occurred first.

### Covariates

Potential confounders were selected a priori based on literature review and risk factors found in our study populations (16,17). Included were age at diagnosis (continuous), sex, race and ethnicity (Asian, Black, Other [Hispanic and Latino, American Indian, and other racial or ethnic group] and White), smoking status (never, former, current), smoking pack-years (continuous), education (less than high school, high school graduation, vocational education, college, university or higher), alcohol consumption (none, moderate drinking up to 1 and 2 drinks per day, heavy drinking >1 and >2 drinks per day for women and men, respectively; 1 drink = 14 grams of ethanol), history of diabetes (yes, no), body mass index (BMI; <18.5, 18.5-24.9, 25.0-29.9, and ≥30.0 kg/m^2^), and hormone therapy in women (yes, no)—all of which were assessed at LTPA assessment. Missing covariates were independently imputed by cohort (16). Established prognostic factors were further included: tumor stage (localized, regional, distant, unknown), histological type (adenocarcinoma, squamous cell carcinoma, other non-small cell carcinoma, small cell carcinoma, unspecified or unknown), and grade (well, moderately, and poorly differentiated; undifferentiated; unknown).

### Statistical Analysis

Using the life-table method and log-rank test, we assessed 5-year relative survival rates by baseline characteristics and clinical features of cancer. Cox proportional hazard regression was used to estimate multivariable-adjusted hazard ratios (HRs) and 95% confidence intervals (CIs) for all-cause and lung cancer–specific mortality, using inactivity as the reference. The global goodness-of-fit test with Schoenfeld residuals found no violation of the proportional hazard assumption. For lung cancer–specific mortality, death from other causes was treated as a competing risk. Given inter- and intrastudy variability, Cox models were stratified by cohort, calendar years of lung cancer diagnosis, and time interval between LTPA assessment and diagnosis. A random-effects meta-analysis was complimented with *I^2^* and *P*_heterogeneity_ to offset potential concerns of residual heterogeneity.

Stratified analyses were conducted by major prognostic and risk factors; to avoid reverse causality due to high fatality and short survival time of distant stage cancer, these analyses were restricted to early stage lung cancer. Additive interactions were evaluated by the relative excess risk due to interaction (30,31), referring to the excess risk from interaction between prediagnosis LTPA and stratification variables as compared with baseline risk without exposure. *P* values were corrected for multiple comparisons by controlling the false-discovery rate. A series of sensitivity analyses were conducted using another LTPA categorization (cohort- and sex-specific quartiles and common quartiles across total participants), excluding participants with long time intervals between LTPA assessment and cancer diagnosis (over median years), further adjusting for dietary calcium intake statistically significantly associated with lung cancer survival in our populations (16), and excluding 1 cohort at a time from analyses. All procedures were performed using SAS 9.4 (SAS Institute, Inc, Cary, NC), and 2-sided *P*-values less than .05 were considered statistically significant.

## Results

Of the 20 494 incident lung cancer patients, 16 864 patients died, including 13 596 deaths from lung cancer (Table 1); the median survival time was 0.9 years (interquartile range = 0.3-2.7). The mean age at lung cancer diagnosis was 68.5 years. Most patients were ever-smokers (ranging from 82.3% to 94.8% across studies), except for those from the Shanghai Women’s Health Study. About 43% of patients met the minimum recommendation before lung cancer diagnosis. The overall 5-year survival rate was 20.9% (95% CI = 20.3% to 21.5%).

Five-year relative survival rates were higher among patients with high educational attainment, noncurrent smokers, smokers with less than 30 pack-years, nondiabetic patients, and women taking hormone therapy (all *P* < .05); those patients also showed higher proportions of meeting the guidelines. When stratified by histological type, stage, and grade, we observed much lower 5-year survival rates for small cell carcinoma (10.0%), distant stage carcinoma (5.9%), and undifferentiated lung carcinoma (11.8%) (Table 2).

**Table 2.** Leisure-time physical activity and 5-year survival rates among lung cancer patients by baseline characteristics

Characteristics	No. of cases	No. of deaths	Meet the guideline, (%)^a^	Median LTPA (IQR) MET-h/week	5-year survival	
					Rate (95% CI), %	*P* ^b^
Age at diagnosis, y						
<70	10 718	8659	41.4	4.5 (0.3-15.0)	22.3 (21.5 to 23.1)	<.001
≥70	9776	8205	44.0	4.9 (0.9-15.0)	18.9 (18.2 to 19.7)	
Sex						
Men	11 010	9384	45.2	4.5 (1.0-15.0)	17.8 (17.1 to 18.6)	<.001
Women	9484	7480	39.7	4.5 (0.4-15.0)	24.1 (23.2 to 25.0)	
Race and ethnicity						
Asian	1852	1356	29.7	0.0 (0.0-10.5)	21.3 (19.4 to 23.3)	.02
Black	954	716	25.3	0.3 (0.0-9.2)	21.8 (19.1 to 24.5)	
Other	219	186	32.0	2.2 (0.3-10.5)	16.9 (12.2 to 22.2)	
White	17469	14606	45.1	5.2 (1.5-15.0)	20.7 (20.1 to 21.3)	
Education						
≤High school	8552	7091	39.6	4.5 (0.3-15.0)	18.4 (17.5 to 19.2)	<.001
Vocational school/some college	6662	5441	41.5	4.5 (1.5-12.2)	21.7 (20.7 to 22.7)	
≥University graduation	5280	4332	49.0	7.7 (1.5-15.0)	23.2 (22.1 to 24.4)	
Smoking status						
Never	2457	1760	46.0	4.8 (0.2-15.0)	29.4 (27.6 to 31.3)	<.001
Former	8233	6875	48.0	6.8 (1.5-15.0)	21.2 (20.3 to 22.1)	
Current	9804	8229	37.8	4.5 (0.3-13.5)	18.2 (17.5 to 19.0)	
Smoking pack-years in smokers						
<30	5647	4536	48.4	7.5 (1.5-15.0)	21.1 (20.1 to 22.3)	<.001
30-49	6324	5324	42.4	4.5 (0.8-15.0)	19.5 (18.5 to 20.5)	
≥50	6066	5244	37.0	4.5 (0.3-10.5)	18.3 (17.3 to 19.3)	
Alcohol consumption^c^						
None	6216	5051	34.8	4.0 (0.0-10.5)	20.0 (19.0 to 21.1)	.004
Moderate	10 031	8255	46.6	6.0 (1.5-15.0)	21.7 (20.9 to 22.5)	
Heavy	4247	3558	44.9	4.5 (0.9-15.0)	19.4 (18.2 to 20.6)	
Body mass index, kg/m^2^						
<18.5	432	357	32.2	2.3 (0.0-10.5)	17.9 (14.4 to 21.8)	.29
18.5-24.99	8836	7227	44.5	5.1 (0.7-15.0)	21.2 (20.3 to 22.0)	
25.0-29.99	8048	6664	44.1	4.5 (1.3-15.0)	20.3 (19.5 to 21.2)	
≥30.0	3178	2616	35.2	4.5 (0.3-10.5)	20.8 (19.4 to 22.2)	
History of diabetes						
No	18 931	15 493	43.2	4.5 (0.7-15.0)	21.2 (20.6 to 21.8)	<.001
Yes	1563	1371	35.5	4.5 (0.3-10.5)	14.8 (13.1 to 16.6)	
Hormone therapy in women						
No	5548	4420	38.2	4.5 (0.3-14.9)	22.8 (21.7 to 24.0)	<.001
Yes	3936	3060	41.8	4.5 (0.9-15.0)	26.0 (24.6 to 27.4)	
Histological type						
Adenocarcinoma	7543	5753	43.5	4.5 (0.7-15.0)	27.0 (26.0 to 28.0)	<.001
Squamous cell carcinoma	3591	2874	42.4	4.5 (0.6-14.9)	24.8 (23.4 to 26.3)	
Other non-small cell carcinoma	3224	2673	42.5	4.5 (1.1-14.8)	20.5 (19.1 to 22.0)	
Small cell carcinoma	2723	2500	40.7	4.5 (0.5-12.3)	10.0 (8.9 to 11.2)	
Unspecified	3413	3064	42.8	4.5 (0.3-15.0)	11.8 (10.7 to 12.9)	
Tumor stage^d^						
Localized	2776	1482	40.4	4.5 (0.3-12.5)	54.3 (52.3 to 56.2)	<.001
Regional	3347	2776	42.4	4.5 (0.3-14.5)	21.0 (19.6 to 22.4)	
Distant	5566	5206	38.8	4.5 (0.3-10.5)	5.9 (5.3 to 6.5)	
Unknown	8805	7400	45.9	6.1 (1.5-15.0)	20.2 (19.3 to 21.0)	
Tumor grade						
Well differentiated	719	423	40.1	4.5 (1.5-10.5)	46.1 (42.3 to 49.8)	<.001
Moderately differentiated	2183	1521	41.6	4.5 (0.8-10.5)	38.5 (36.4 to 40.5)	
Poorly differentiated	3767	3075	40.3	4.5 (0.7-10.5)	22.5 (21.2 to 23.9)	
Undifferentiated	1296	1200	39.0	4.5 (0.8-10.5)	11.8 (10.1 to 13.6)	
Unknown	12 529	10 645	44.0	4.8 (0.4-15.0)	16.7 (16.0 to 17.3)	
From baseline to diagnosis, y^e^						
<5	7615	6706	40.1	4.5 (0.3-10.5)	19.8 (18.9 to 20.7)	.02
5-9	8559	6942	43.3	4.5 (0.7-14.6)	20.6 (19.8 to 21.5)	
≥10	4320	3216	45.9	7.2 (1.5-18.1)	22.6 (21.3 to 23.9)	

a Percentage of adherence to the recommended physical activity guidelines, ≥ at least 500 MET-minutes (8.3 MET-hours) per week. CI = confidence interval; IQR = interquartile range; LTPA = leisure-time physical activity; MET-h/week = metabolic-equivalent hours per week.

b Statistical differences across survival rates (2-sided *P* values) were estimated by the log-rank test and corrected for multiple comparisons by controlling the false-discovery rate.

c Moderate defined as >0 to ≤1 (women) or >0 to ≤2 (men) drinks per day and heavy defined as >1 (women) or >2 (men) drinks per day.

d Localized, regional, and distant stages included stage I and II, stage III, and stage IV, respectively.

e Time interval from physical activity assessment to lung cancer diagnosis.

Compared with inactivity (Table 3), LTPA of 8.3 MET-h/week or more before cancer diagnosis was associated with a lower hazard of all-cause mortality among the overall population (HR = 0.93, 95% CI = 0.88 to 0.99), but not with lung cancer–specific mortality (HR = 0.99, 95% CI = 0.95 to 1.04), after adjustment for all potential covariates. No heterogeneity was observed across cohorts (*P*_heterogeneity_ = .41 for all-cause and .28 for lung cancer–specific mortality), with comparable HRs from random-effects meta-analyses of 0.95 (95% CI = 0.90 to 1.00) and 1.00 (95% CI = 0.95 to 1.05), respectively (Supplementary Figures 1 and 2, available online). Clear evidence of additive interaction was found by stage (*P*_interaction_ = .008 for all-cause mortality and .003 for lung cancer–specific mortality). For localized lung cancer, prediagnosis LTPA of at least 8.3 MET-h/week was associated with 20% lower mortality (HR = 0.80, 95% CI = 0.67 to 0.97, for all-causes, and HR = 0.80, 95% CI = 0.65 to 0.99, for lung cancer). Meanwhile, overall associations were weaker for regional stage lung cancer (HR = 0.89, 95% CI = 0.77 to 1.02, and HR = 0.95, 95% CI = 0.84 to 1.07, respectively), and null associations were found for distant stage (Table 3). Results from random-effects meta-analyses yielded similar results as those presented, with about 20% lower mortality among localized cases who adhered to prediagnosis LTPA of at least 8.3 MET-h/week (HR = 0.80, 95% CI = 0.66 to 0.98, for all-cause mortality; *P*_heterogeneity_ = .48; and HR = 0.76, 95% CI = 0.61 to 0.94, for lung cancer–specific mortality; *P*_heterogeneity_ = .50; Supplementary Figures 3 and 4, available online). Exclusion of any cohort from the main analysis one at a time had limited impacts on the above-reported associations (Supplementary Table 2, available online).

**Table 3.** Association of prediagnosis leisure-time physical activity with all-cause and lung cancer–specific mortality among lung cancer patients by tumor stage^a^

Tumor stage	Leisure-time physical activity (MET-h/week)^b^							
	Deaths from all causes				Deaths from lung cancer^c^			
	None	>0 to <8.3	≥8.3	*P*_trend_ ^d^	None	>0 to <8.3	≥8.3	*P*_trend_ ^d^
Total cases								
Deaths/cases, No.	2380/3008	7297/8749	7187/8737		1965/2822	5764/8267	5867/8328	
5-year survival rate (95% CI), %	18.1 (16.7 to 19.6)	21.1 (20.2 to 22.0)	21.2 (20.3 to 22.1)		23.7 (22.0 to 25.4)	28.5 (27.5 to 29.5)	27.4 (26.4 to 28.4)	
Hazard ratio (95% CI)^e^	1 (Referent)	0.96 (0.91 to 1.01)	0.92 (0.87 to 0.97)	.002	1 (Referent)	0.99 (0.94 to 1.04)	0.98 (0.93 to 1.02)	.29
Hazard ratio (95% CI)^f^	1 (Referent)	0.97 (0.91 to 1.02)	0.93 (0.88 to 0.99)	.01	1 (Referent)	1.00 (0.96 to 1.05)	0.99 (0.95 to 1.04)	.63
Localized lung cancer cases								
Deaths/cases, No.	226/485	654/1170	602/1121		165/473	391/1152	405/1097	
5-year survival rate (95% CI), %	52.9 (47.9 to 57.6)	54.3 (51.3 to 57.3)	54.8 (51.7 to 57.8)		60.1 (55.0 to 64.9)	66.2 (63.2 to 69.1)	64.3 (61.2 to 67.2)	
Hazard ratio (95% CI)^e^	1 (Referent)	0.92 (0.77 to 1.10)	0.79 (0.66 to 0.95)	.009	1 (Referent)	0.84 (0.68 to 1.03)	0.80 (0.65 to 0.99)	.42
Hazard ratio (95% CI)^f^	1 (Referent)	0.93 (0.78 to 1.12)	0.80 (0.67 to 0.97)	.02	1 (Referent)	0.84 (0.68 to 1.04)	0.80 (0.65 to 0.99)	.46
Regional lung cancer cases								
Deaths/cases, No.	397/510	1176/1419	1203/1418		332/480	945/1377	996/1353	
5-year survival rate (95% CI), %	20.4 (16.9 to 24.1)	22.3 (20.1 to 24.5)	19.8 (17.8 to 22.0)		25.6 (21.5 to 29.8)	29.5 (27.1 to 32.1)	25.4 (23.0 to 27.8)	
Hazard ratio (95% CI)^e^	1 (Referent)	0.94 (0.82 to 1.08)	0.87 (0.76 to 1.00)	.06	1 (Referent)	0.98 (0.86 to 1.11)	0.93 (0.83 to 1.05)	.42
Hazard ratio (95% CI)^f^	1 (Referent)	0.94 (0.82 to 1.09)	0.89 (0.77 to 1.02)	.17	1 (Referent)	0.98 (0.86 to 1.11)	0.95 (0.84 to 1.07)	.51
Distant lung cancer cases								
Deaths/cases, No.	995/1093	2174/2315	2037/2158		885/1048	1900/2252	1815/2113	
5-year survival rate (95% CI), %	6.3 (4.9 to 7.8)	5.6 (4.7 to 6.6)	6.0 (5.1 to 7.1)		10.0 (8.2 to 12.0)	10.7 (9.4 to 12.1)	10.4 (9.1 to 11.8)	
Hazard ratio (95% CI)^e^	1 (Referent)	0.98 (0.89 to 1.07)	0.97 (0.89 to 1.06)	.61	1 (Referent)	1.03 (0.97 to 1.10)	1.04 (0.98 to 1.10)	.50
Hazard ratio (95% CI)^f^	1 (Referent)	0.98 (0.90 to 1.08)	0.98 (0.89 to 1.07)	.73	1 (Referent)	1.03 (0.97 to 1.10)	1.04 (0.98 to 1.11)	.53

a Localized, regional, and distant stages included stage I and II, stage III, and stage IV, respectively. Additive interactions were statistically significant: *P* interaction = .008 for all-cause mortality and .003 for lung cancer–specific mortality. CI = confidence interval; MET-h/week = metabolic-equivalent hours per week.

b ≥500 MET-min/wk (≥8.3 MET-h/week) was the level recommended for substantial health benefits based on the physical active guidelines such as the World Health Organization Global Recommendations and 2018 Physical Activity Guidelines.

c Patients missing cause of death were excluded from the analysis; death from other causes was treated as a competing risk.

d Corrected for multiple comparisons by controlling the false-discovery rate.

e Adjusted for age at diagnosis, sex, smoking status, and smoking pack-years and stratified by cohort, year of lung cancer diagnosis, and time interval from leisure-time physical activity assessment to lung cancer diagnosis.

f Adjusted for age at diagnosis, sex, smoking status, smoking pack-years, race and ethnicity, education, alcohol consumption, history of diabetes, body mass index levels, hormone therapy in women, and histological type and grade of lung cancer and stratified by cohort, year of lung cancer diagnosis, and time interval from leisure-time physical activity assessment to lung cancer diagnosis.

**Figure 1.**
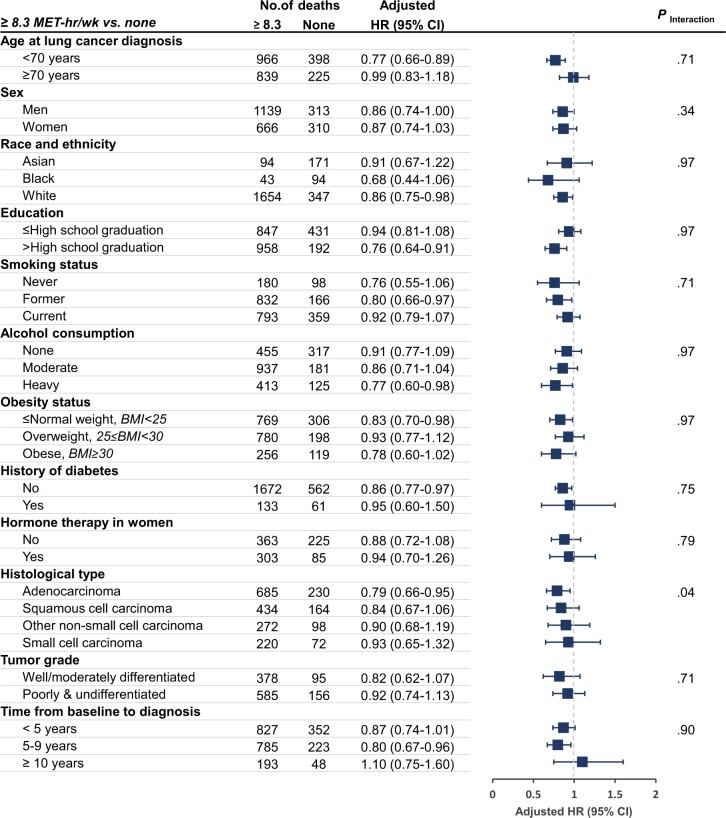
Prediagnosis leisure-time physical activity and all-cause mortality among lung cancer patients: stratified analyses of localized and regional stage cases. HRs (95% CIs) for ≥8.3 MET-h/week vs none were shown after adjusting for age at diagnosis, sex, smoking status, smoking pack-years, race and ethnicity, education, alcohol consumption, history of diabetes, BMI levels, hormone therapy in women, histological type, tumor stage, and grade of lung cancer and stratifying by cohort, year of lung cancer diagnosis, and time interval from leisure-time physical activity assessment to lung cancer diagnosis. Interaction (additive) refers to global *P* value for relative excess risk due to interaction between prediagnosis leisure-time physical activity and each stratification variable. All *P* values were corrected for multiple comparisons by controlling the false-discovery rate. All statistical tests were 2-sided. Error bars represent the 95% CIs. BMI = body mass index; CI = confidence interval; HR = hazard ratio; MET-h/week = metabolic-equivalent hours per week.

**Figure 2.**
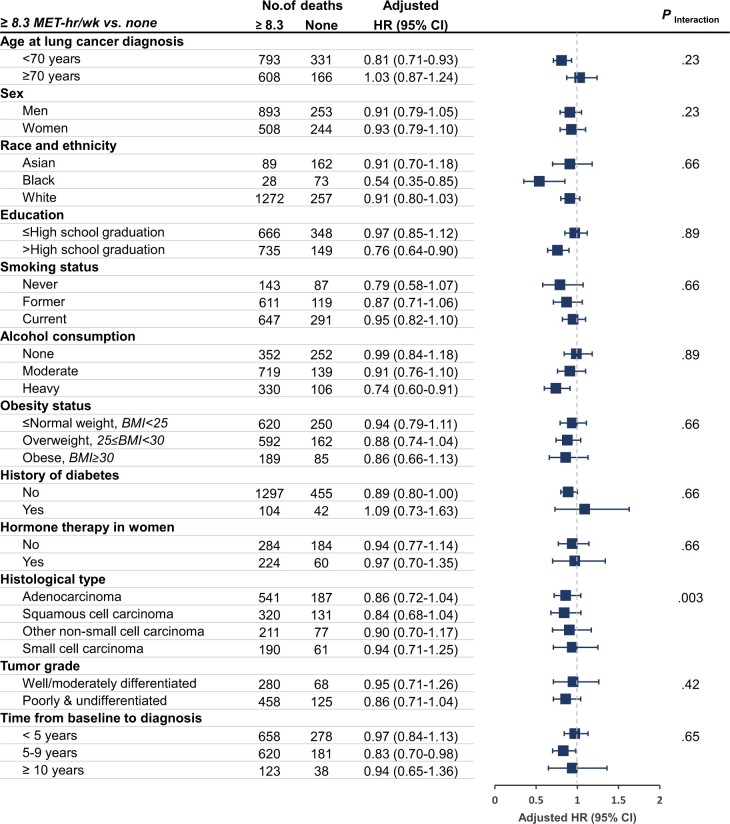
Prediagnosis leisure-time physical activity and lung cancer–specific mortality among lung cancer patients: stratified analyses of localized and regional stage cases. HRs (95% CIs) for ≥8.3 MET-h/week vs none were shown after adjusting for age at diagnosis, sex, smoking status, smoking pack-years, race and ethnicity, education, alcohol consumption, history of diabetes, BMI levels, hormone therapy in women, histological type, tumor stage, and grade of lung cancer and stratifying by cohort, year of lung cancer diagnosis, and time interval from leisure-time physical activity assessment to lung cancer diagnosis. For the lung-cancer mortality analyses, cases missing cause of death were excluded from the analysis, and death from other causes was treated as a competing risk. Interaction (additive) refers to global *P* value for relative excess risk because of interaction between prediagnosis leisure-time physical activity and each stratification variable. All *P* values were corrected for multiple comparisons by controlling the false-discovery rate. All statistical tests were 2-sided. Error bars represent the 95% CIs. BMI = body mass index; CI = confidence interval; HR = hazard ratio; MET-h/week = metabolic-equivalent hours per week.

In stratified analyses of localized and regional-stage lung cancer (Figures 1 and 2), we observed additive interactions when jointly considering prediagnosis LTPA and histological type (*P*_interaction_ = .04 for all-cause mortality and .003 for lung cancer–specific mortality). Prediagnosis LTPA of at least 8.3 MET-h/week was associated with 21% lower all-cause mortality for adenocarcinoma (HR = 0.79, 95% CI = 0.66 to 0.95). The overall association pattern remained consistent across the potential prognostic and risk factors (eg, race and ethnicity and BMI; all *P*_interaction_ > .05), but the magnitude of the associations attenuated when LTPA assessment was far from cancer diagnosis. Notably, never-smokers also appeared to have survival benefits from prediagnosis LTPA of at least 8.3 MET-h/week (HR = 0.76, 95% CI = 0.55 to 1.06, for all-cause mortality, and HR = 0.79, 95% CI = 0.58 to 1.07, for lung cancer–specific mortality), but the point estimates failed to reach statistical significance because of the small sample size.

A series of sensitivity analyses showed a similar pattern of the associations (data not shown). Further analyses separating the moderately active (8.3-16.0 MET-h/week) and the highly active (≥16.0 MET-h/week) groups showed little evidence that the latter was more strongly related to lung cancer survival; however, the risk estimate was unstable because of its insufficient sample size (data not shown).

## Discussion

In this pooled analysis of 20 494 incident lung cancer patients from 11 prospective cohorts, regular participation in LTPA prior to cancer diagnosis, particularly when meeting or exceeding the minimum Physical Activity Guidelines, was associated with reduced hazards of mortality among lung cancer patients. Notably, patients diagnosed with localized lung cancer showed approximately 20% lower all-cause and lung cancer–specific mortality when engaging in LTPA of at least 8.3 MET-h/week before diagnosis compared with inactivity. A statistically significant additive interaction by stage was suggested. Our findings support a possible long-term benefit of habitual LTPA adhering to the Physical Activity Guidelines. If confirmed, pretreatment LTPA could be proposed as a possible stratification factor for future therapeutic trials, at least for early stage lung cancer patients.

Currently, epidemiological evidence on survival benefits attributed to prediagnosis LTPA remains limited among lung cancer patients (8,9,11,12). In a recent systematic review and meta-analysis of all existing epidemiologic studies and trials (7), the summary HR for lung cancer–specific mortality associated with higher levels of prediagnosis PA was 0.81 (95% CI = 0.75 to 0.87); no data was available for all-cause mortality. Despite adding an important piece of evidence, this finding was derived as a part of 5 studies accessing multiple cancer sites, not focusing only on lung cancer. Thus, overall numbers of lung cancer patients and deaths were limited, and lung cancer–specific prognostic and risk factors could not be properly considered. Several observational studies also showed the beneficial impact of prediagnosis PA on lung cancer mortality (13-15), in line with our findings. For example, the β-Carotene and Retinol Efficacy Trial, including 231 lung cancer cases and 141 deaths, found an inverse association of prediagnosis total PA with mortality only among women who smoked heavily (13). The Women’s Health Initiative study, analyzing 2148 lung cancer patients and 1 365 lung cancer deaths, reported 20%-32% lower lung-cancer mortality associated with at least 100 MET-minutes/week of exercise among postmenopausal women (14), with a dose-response association for adenocarcinoma only. Recently, a hospital-based, case-control study (579 cases with 560 total deaths and 481 lung cancer–specific deaths) reported that lifetime recreational physical inactivity was associated with 30%-40% increased mortality (HR = 1.31, 95% CI = 1.09 to 1.58, for all-cause mortality, and HR = 1.40, 95% CI = 1.14 to 1.71, for lung cancer–specific mortality) (15). Consistent with our findings, this study observed a stronger inactivity–mortality association among early stage lung cancer cases; no association was found for distant stage. Similarly, some cohort studies showed that higher cardiorespiratory fitness levels prior to cancer diagnosis were linked to a lower risk of death among lung cancer patients (32-35). Nonetheless, most previous studies were limited to insufficient sample size, restricted to predominantly White populations, and lacked consideration of major prognostic factors, subgroup variations, and potential competing risks of death. Our large sample size, including 20 494 incident lung cancer patients with different stage and histology and 16 864 deaths from racially and ethnically diverse populations, detailed data on clinical features of lung cancer, and enhanced scientific rigor would overcome the previous limitations. Findings from this pooled analysis suggest that the association of prediagnosis LTPA with lung cancer survival could be modified by tumor stage and histological type. Black patients appeared to have lower HRs than others, although the test for interaction was not statistically significant. Also, the overall associations remained consistent across sex, smoking, BMI, and other factors potentially related to survival, adding a piece of epidemiologic evidence to a possible causal association.

PA has attracted much attention as a potential protective factor against cancer-related deaths, recurrence, or metastasis (4-6,36). During exercise, the body activates biological mechanisms which inhibit tumor growth, including modulation of carcinogenic factors (ie, inflammatory cytokines, insulin-like growth factors, and other hormones) and enhancement in immune function and metabolic health (6). Long-term habitual PA can lead to intratumoral adaptations, including improvements in blood perfusion, immunogenicity, and immune cell infiltration (4,5), which help inhibit cancer progression. Given these biological benefits, it is possible that PA before initiation of carcinogenesis may result in developing less aggressive tumors. Furthermore, PA plays a crucial role in enhancing drug tolerance and efficacy, alleviating treatment-related adverse effects, and reducing the likelihood of relapse and metastasis (4,36). Regarding lung-specific benefits from PA, evidence indicates that greater amounts of PA are associated with less lung function decline, better pulmonary functional capacity, and a lower risk of chronic obstructive pulmonary disease (37-39). Indeed, a recent systematic review of randomized-controlled trials has reported that presurgery exercise interventions could substantially improve physical and pulmonary functions among lung cancer patients and reduce postsurgery complications (40). In our study, the association of prediagnosis LTPA appeared to be stronger when exposure was measured closer to diagnosis, lending some support to the mechanisms mentioned above. Existing biological evidence and our epidemiological observations suggest that habitual LTPA may improve lung cancer survivorship, especially for early stage lung cancer. However, we did not find any statistically significant association with distant stage cancer. We speculate that the high fatality rate and short survival time for late stage lung cancer made it difficult for us to detect a moderate association with prediagnosis LTPA, if one exists.

Our study has several strengths. This is the largest prospective investigation on the association of prediagnosis LTPA with lung cancer survival. We used individual participant data of more than 20 000 incident lung cancer patients from diverse populations. Our prospective design, large sample size, and extensive information on a wide range of clinical characteristics, smoking history, and other lifestyle factors enabled comprehensive analyses. All the analyses were controlled for or stratified by major prognostic and risk factors and considered potential competing risks of death, which enhanced the scientific rigor of our study. Nonetheless, several limitations should be acknowledged. First, because of a lack of information, we could not control for the influence of lung cancer treatment and care in the current study. To compensate for this limitation, we applied statistical models stratified by calendar year at lung cancer diagnosis with adjustment for treatment-related clinic factors (ie, histological type, stage, and grade). Second, we used a one-time measure of prediagnosis LTPA; thus, we could not consider changes in LTPA intensity or patterns over time, as well as other domains of PA (ie, occupation, household, and transportation). Measurement errors in self-reports (eg, overreporting) and MET estimations based on varying instruments across cohorts are another concern, despite using validated questionnaires (18-26). Our findings might be somewhat affected by these exposure misclassifications. Third, postdiagnosis information was unavailable in most participating studies. Given that the mean survival time after lung cancer diagnosis is relatively short, however, it may be challenging to find the actual impact of postdiagnosis factors, including LTPA, on lung cancer survival in a population-based setting. Furthermore, LTPA assessed after diagnosis would be more likely affected by diseases, resulting in bias because of reverse causation. Fourth, despite carefully adjusting for both smoking status and lifetime tobacco exposure in all analyses, residual confounding by smoking cannot be completely ruled out. For example, it is possible that heavy smokers are less likely to engage in LTPA and also more likely to underreport their smoking exposure. Finally, insufficient sample size of some subgroup analyses (eg, never-smokers, Black, and Asian participants) might contribute to the failure of reaching statistical significance in some of the observed associations.

This large-scale pooled analysis of 11 cohorts indicates that regular participation in LTPA, particularly when meeting or exceeding the minimum Physical Activity Guidelines, is associated with reduced mortality among lung cancer patients, especially those with early stage cancer. Our findings add the supporting evidence that adhering to the Physical Activity Guidelines before cancer diagnosis may have long-term benefits on lung cancer progression and/or survival. Future investigation incorporating various domains of PA objectively assessed at multiple time points throughout the life course and the lung cancer continuum is needed to confirm certain benefits of PA and develop PA promotion strategies for reducing the global burdens of lung cancer.

## Funding

This work was partially supported by a grant from the National Institutes of Health (R03 CA183021) and by the Ingram Cancer Professorship fund to Dr XO Shu. HPFS and NHS were supported by grants from the National Institutes of Health (HPFS: U01 CA167552; NHS: UM1 CA186107 and P01 CA87969).

## Notes

**Role of the funders:** The funders had no role in the design and conduct of the study; the collection, management, analysis, or interpretation of the data; preparation, review, or approval of the manuscript; or the decision to submit the manuscript for publication.

**Disclosures:** All authors report no conflicts of interest.

**Author contributions:** Conceptualization and Methodology: XOS, JJY, DY. Data curation and Resources: EW, DHL, WB, KR, RS, YP, YT, YTG, EMM, RK, AL, KBB, QL, EPS, XZ, CZ, KS-B, LAS, MDC, GS, KO, CS, DA, MJ, SAS-W, WZ, XOS. Formal analysis and Visualization: JJY, DY, XOS. Writing—original draft: JJY, XOS. Writing—review & editing: All authors. Supervision: XOS.

**Acknowledgements:** The data used for this study were contributed by the National Cancer Institute Cohort Consortium. The authors want to thank staff, investigators, and participants of the contributing cohorts. We thank Dr. Mary Shannon Byers for her assistance in editing and preparing the manuscript. MJ and KS-B are identified as personnel of the International Agency for Research on Cancer/World Health Organization. These authors alone are responsible for the views expressed in this article, and they do not necessarily represent the decisions, policy, or views of the International Agency for Research on Cancer/World Health Organization.

The HPFS and NHS study protocols were approved by the institutional review boards of the Brigham and Women’s Hospital and Harvard T.H. Chan School of Public Health and those of participating registries as required. We acknowledge Channing Division of Network Medicine, Department of Medicine, Brigham and Women’s Hospital as home of the NHS. We also would like to thank the participants and staff of the HPFS and NHS for their valuable contributions as well as the following state cancer registries for their help: AL, AZ, AR, CA, CO, CT, DE, FL, GA, ID, IL, IN, IA, KY, LA, ME, MD, MA, MI, NE, NH, NJ, NY, NC, ND, OH, OK, OR, PA, RI, SC, TN, TX, VA, WA, WY. The authors assume full responsibility for analyses and interpretation of these data.

## Data Availability

The data underlying this article will be shared on reasonable request to the corresponding author after approval of principle investigators of participating cohorts.

## Supplementary Material

pkac009_Supplementary_Data
