# Supplementary material for: Prediagnosis Leisure-Time Physical Activity and Lung Cancer Survival: A Pooled Analysis of 11 Cohorts
Source: JNCI Cancer Spectr. 2022 Feb 14;6(2):pkac009. doi: 10.1093/jncics/pkac009 (PMC8962711; doi:10.1093/jncics/pkac009)
Supplement: pkac009_Supplementary_Data [file pkac009_supplementary_data.pdf]

Supplementary Material

Pre-diagnosis Leisure-Time Physical Activity and Lung Cancer Survival: A Pooled Analysis of 11 Cohorts.

Jae Jeong Yang, Danxia Yu, Emily White, Dong Hoon Lee, William Blot, Kim Robien, Rashmi Sinha, Yikyung Park, Yumie Takata, Yu-Tang Gao, Karl Smith-Byrne, Evelyn M. Monninkhof, Rudolf Kaaks, Arnulf Langhammer, Kristin Benjaminsen Borch, Laila Al-Shaar, Qing Lan, Elin Pettersen Sørgjerd, Xuehong Zhang, Clair Zhu, María Dolores Chirlaque, Gianluca Severi, Kim Overvad, Carlotta Sacerdote, Dagfinn Aune, Mattias Johansson, Stephanie A. Smith-Warner, Wei Zheng, Xiao-Ou Shu.

Supplementary Table 1. Details about exposure (leisure-time physical activity) assessment in each cohort

Cohort Name (Abbreviation)	Questions About LTPA	Questions About LTPA Time	Exposure Window	Intensity
National Health Institute-AARP Diet and Health Study (AARP)	During a typical month in the past 12 months, how often did you participate in physical activities, including exercise, sports, and activities such as carrying heavy loads? Only include periods of physical activities that lasted at least 20 minutes and causes increases in breathing or heart rate or causes you to work up a sweat.	Frequency<br>Never<br>Rarely<br>1-3 times/month<br>1-2 times/week<br>3-4 times/week<br>≥5 times/week	One year	Using the absolute MET intensity<br>4.0 MET for moderate activities
Health Professionals Follow-up Study (HPFS)	During the past year, what was your average time per week at each activity?<br>Walking or hiking outdoors (including walking at golf)/<br>Jogging (slower than 10 min/mile)<br>Running (slower than 10 min/mile or faster)<br>Bicycling (including stationary machine)<br>Lap swimming<br>Tennis<br>Squash or racket ball<br>Calisthenics or rowing	Average total time per week<br>None<br>1-4 min<br>5-19 min<br>20-39 min<br>40-90 min<br>1.5 hrs.<br>2-3 hrs.<br>4-6 hrs.<br>7-10 hrs.<br>≥11 hrs.	One year	A compendium of physical activity was used to assign METs for each activity.
Nurses' Health Study (NHS)	During the past year, what was your average time per week spent at each of the following activities:<br>Walking or hiking outdoors (including walking at golf)<br>Jogging (slower than 10 min/mile)<br>Running (slower than 10 min/mile or faster)<br>Bicycling (including stationary machine)<br>Lap swimming<br>Tennis<br>Calisthenics / aerobics / aerobic dance / rowing machine<br>Squash or racket ball	Average total time per week<br>Zero<br>1-4 min<br>5-19 min<br>20-59 min<br>1 hr.<br>1-1.5 hrs.<br>2-3 hrs.<br>4-6 hrs.<br>7-10 hrs.<br>≥11 hrs.	One year	A compendium of physical activity was used to assign METs for each activity.
Iowa Women's Health Study (IWHS)	Aside from any work you do at home or at a job, do you do anything regularly—that is on a daily basis—that helps you keep physically fit?<br>How often, in your free time, do you take part in moderate physical activity (such as	Frequency<br>more than 4 times a week<br>2-4 times a week<br>about once a week<br>a few times a month<br>a few times a year	Not specified<br>Regular daily basis	Using the absolute MET intensity<br>4.0 MET for moderate activities<br>7.0 MET for hard physical activities

	bowling, golf, light sports or physical exercise, gardening, taking long walks)?<br>How often, in your free time, do you take part in vigorous physical activity (such as jogging, racquet sports, swimming, aerobics, strenuous sports)?	rarely or never		
Prostate, Lung, Colorectal and Ovarian Cancer Screening Trial (PLCO)	Over the last 12 months, on average, how many days per week did you spend in any physical activity strenuous enough to work up a sweat or to increase your breathing and heart rate to very high levels?<br>Over the last 12 months, on average, how many days per week did you spend in any moderate physical activity where you worked up a light sweat or increased your breathing and heart rate to moderately high levels?<br>None or < 1 Day/Week<br>2-3 Days/Week<br>4-5 Days/Week<br>6-7 Days/Week	Over the last 12 months, on average, how long was each session of strenuous activity?<br>Over the last 12 months, on average, how long was each session of moderate activity?<br>None or less than 15 minutes<br>16 to 19minutes<br>20 to 29 minutes<br>30 to 39 minutes<br>40 minutes or more	One year	Using the absolute MET intensity<br>4.0 MET for moderate activities<br>7.0 MET for hard physical activities
Southern Community Cohort Study (SCCS)	How much time per week do you typically spend doing?<br>Moderate sports such as bowling, dancing, golfing, or softball<br>Vigorous sports such as jogging, aerobics, bicycling, tennis, swimming, weightlifting, or basketball	Total time (continuous)<br>Hours<br>Minutes	One year	A compendium of physical activity was used to assign METs for each activity.
VITamins And Lifestyle Study (VITAL)	In the past 10 years, did you walk for exercise, including walking on a treadmill?<br>Usual pace (casual: each mile takes ≥30 min., moderate: each mile takes 20-29 min., fast: each mile takes ≤19 min.)<br>In the past 10 years, did you lift weights or use weight machines?<br>In the past 10 years, did you do yoga?<br>In the past 10 years, did you do mild exercise such as golf, slow dancing, or bowling?<br>In the past 10 years, did you do moderate or strenuous exercise such as running, aerobics, folk dancing, swimming, cycling, or sports? What types of exercise did you do most often?	How many years in past 10 years?<br>Days per week?<br>Minutes per day?<br>Hours per day?	10 years	A compendium of physical activity was used to assign METs for each activity.
European Prospective Investigation into Cancer & Nutrition (EPIC)	In a typical week during the past year, how many hours did you spend per week on each of the following activities?	Hours per week	One year	Using the EPIC data manual guidelines<br>3.0 MET for walking

	walking, including walking to work, shopping and leisure time (summer/winter)<br>cycling, including cycling to work, shopping and leisure time (summer/winter)<br>physical exercise such as fitness, aerobics, swimming, jogging, tennis, etc.<br>In a typical week during the past year, did you engage in any of these activities vigorously enough to cause sweating or faster heartbeat?			6.0 MET for cycling and sports<br>8.0 MET for stair climbing
Trøndelag Health Study (HUNT)	How has your leisure-time physical activity been the last year? Think of a weekly average for the year.<br>Light activity: no sweating or being out of breath<br>Hard physical activity: sweating out of breath	Hours per week<br>None<br>Under 1<br>1-2<br>3 and more	One year	Using the absolute MET intensity<br>3.0 MET for light activities<br>7.0 MET for hard physical activities
Shanghai Men's Health Study (SMHS)	Over the past 5 year, how often did you attend exercise? (The often means once a week at least, for 3 months continuously)<br>Please tell me which activity did you do most time during that period? 3 kinds of exercises.<br>Please measure your activity level when you took exercise: 0 indicates nothing at all, 10 indicates very very strong.	How many hours per week?<br>How many years did you participate?	5 years	A compendium of physical activity was used to assign METs for each activity.
Shanghai Women's Health Study (SWHS)	Over the past five years, have you participated in any sports activities often? ("often" means at least once a week, for more than 3 months, continuously)<br>Please tell me of 3 sports activities you most often participated in during this period.<br>When you do exercises, do you:<br>sweat every time<br>sweat most of the times<br>normally do not sweat	How many hours did you spend each week? (If it is less than 1 hour, fill in <1)<br>How many years have you participated in each of the activities?	5 years	A compendium of physical activity was used to assign METs for each activity.

Supplementary Table 2. Hazard ratios (95% CIs) [a, b] for all-cause and lung-cancer specific mortality associated with pre-diagnosis leisure-time physical activity: Excluding one cohort at a time from the main analysis

Cohort excluded	Leisure-time Physical Activity (MET-h/week) [c]							
	Deaths from all causes				Deaths from lung cancer [d]			
	None	>0 to < 8.3	≥ 8.3	P_{trend} [b,e]	None	>0 to < 8.3	≥ 8.3	P_{trend} [b,e]
Localized lung cancer cases								
All cohort combined	1 (ref.)	0.93 (0.78-1.12)	0.80 (0.67-0.97)	0.004	1 (ref.)	0.84 (0.68-1.04)	0.80 (0.65-0.99)	0.16
Excluding AARP	1 (ref.)	0.94 (0.71-1.23)	0.79 (0.60-1.02)	0.06	1 (ref.)	0.93 (0.70-1.25)	0.83 (0.62-1.12)	0.22
Excluding HPFS	1 (ref.)	0.93 (0.78-1.12)	0.80 (0.67-0.97)	0.004	1 (ref.)	0.84 (0.68-1.04)	0.80 (0.65-0.99)	0.16
Excluding NHS	1 (ref.)	0.93 (0.78-1.12)	0.80 (0.67-0.97)	0.004	1 (ref.)	0.84 (0.68-1.04)	0.80 (0.65-0.99)	0.16
Excluding IWHS	1 (ref.)	0.89 (0.73-1.08)	0.76 (0.63-0.93)	0.003	1 (ref.)	0.81 (0.65-1.00)	0.76 (0.61-0.95)	0.11
Excluding PLCO	1 (ref.)	0.93 (0.68-0.98)	0.81 (0.68-0.98)	0.009	1 (ref.)	0.84 (0.69-1.04)	0.81 (0.65-1.00)	0.20
Excluding SCCS	1 (ref.)	0.95 (0.79-1.14)	0.82 (0.68-0.99)	0.005	1 (ref.)	0.86 (0.70-1.07)	0.82 (0.66-1.02)	0.21
Excluding VITAL	1 (ref.)	0.96 (0.78-1.16)	0.84 (0.69-1.02)	0.02	1 (ref.)	0.86 (0.69-1.07)	0.83 (0.67-1.04)	0.33
Excluding EPIC	1 (ref.)	0.95 (0.78-1.14)	0.80 (0.66-0.97)	0.003	1 (ref.)	0.86 (0.69-1.07)	0.82 (0.65-1.01)	0.20
Excluding HUNT	1 (ref.)	0.93 (0.78-1.12)	0.80 (0.67-0.97)	0.004	1 (ref.)	0.84 (0.68-1.04)	0.80 (0.65-0.99)	0.16
Excluding SMHS	1 (ref.)	0.91 (0.76-1.11)	0.78 (0.64-0.95)	0.002	1 (ref.)	0.80 (0.64-0.99)	0.75 (0.60-0.94)	0.11
Excluding SWHS	1 (ref.)	0.95 (0.78-1.14)	0.81 (0.67-0.99)	0.006	1 (ref.)	0.82 (0.66-1.01)	0.78 (0.62-0.97)	0.15

[a]Localized and regional stages included stage I and II, and stage III, respectively. Abbreviations: AARP, National Health Institute-AARP Diet and Health Study; HPFS, Health Professionals Follow-up Study; NHS, Nurses' Health Study; IWHS, Iowa Women's Health Study; PLCO, Prostate, Lung, Colorectal and Ovarian Cancer Screening Trial; SCCS, Southern Community Cohort Study; VITAL, VITamins And Lifestyle Study; EPIC, European Prospective Investigation into Cancer & Nutrition; HUNT, Trøndelag Health Study; SMHS, Shanghai Men's Health Study; SWHS, Shanghai Women's Health Study; MET-hours/week, metabolic equivalent hours per week; CI, confidence interval;

[b]Adjusted for age at diagnosis, sex, smoking status, smoking pack-years, race and ethnicity, education, alcohol consumption, history of diabetes, body mass index levels, hormone therapy in women, histological type, and grade of lung cancer; and stratified by cohort, year of lung cancer diagnosis, and time interval from leisure-time physical activity assessment to lung cancer diagnosis.

[c]≥500 MET-minutes/week (≥8.3 MET-hours/week) was the level recommended for substantial health benefits based on the physical activity guidelines such as WHO Global Recommendations and 2018 Physical Activity Guidelines.

[d]Cases who were missing cause of death were excluded from the analysis; death from other causes was treated as a competing risk.

[e] Linear trends were tested using median values of each category—all statistical tests were 2-sided.

Cohort	Country	No. Cases	No. Deaths	≥8.3 MET-hr/wk (%)	Adjusted HR (95% CI)	Weight (%)
**AARP**	USA	9684	8407	39.2	0.87 (0.79, 0.94)	32.71
**HPFS**	USA	983	885	51.5	0.83 (0.36, 1.88)	0.42
**NHS**	USA	1542	1257	40.4	1.04 (0.93, 1.17)	19.12
**IWHS**	USA	1017	914	41.9	0.97 (0.79, 1.21)	6.13
**PLCO**	USA	666	263	34.1	0.96 (0.73, 1.27)	3.63
**SCCS**	USA	815	585	17.2	0.98 (0.77, 1.24)	4.98
**VITAL**	USA	1029	764	32.7	0.97 (0.79, 1.18)	6.89
**EPIC**	Europe	2540	2081	81.5	0.90 (0.75, 1.07)	8.46
**HUNT**	Europe	474	432	25.5	1.04 (0.73, 1.47)	2.35
**SMHS**	Asia	918	706	30.3	1.10 (0.92, 1.31)	8.63
**SWHS**	Asia	826	570	26.4	0.89 (0.72, 1.09)	6.68
**Overall (*I*-squared = 3.7%, *P*-heterogeneity=0.41)**					**0.95 (0.90, 1.00)**	**100.00**

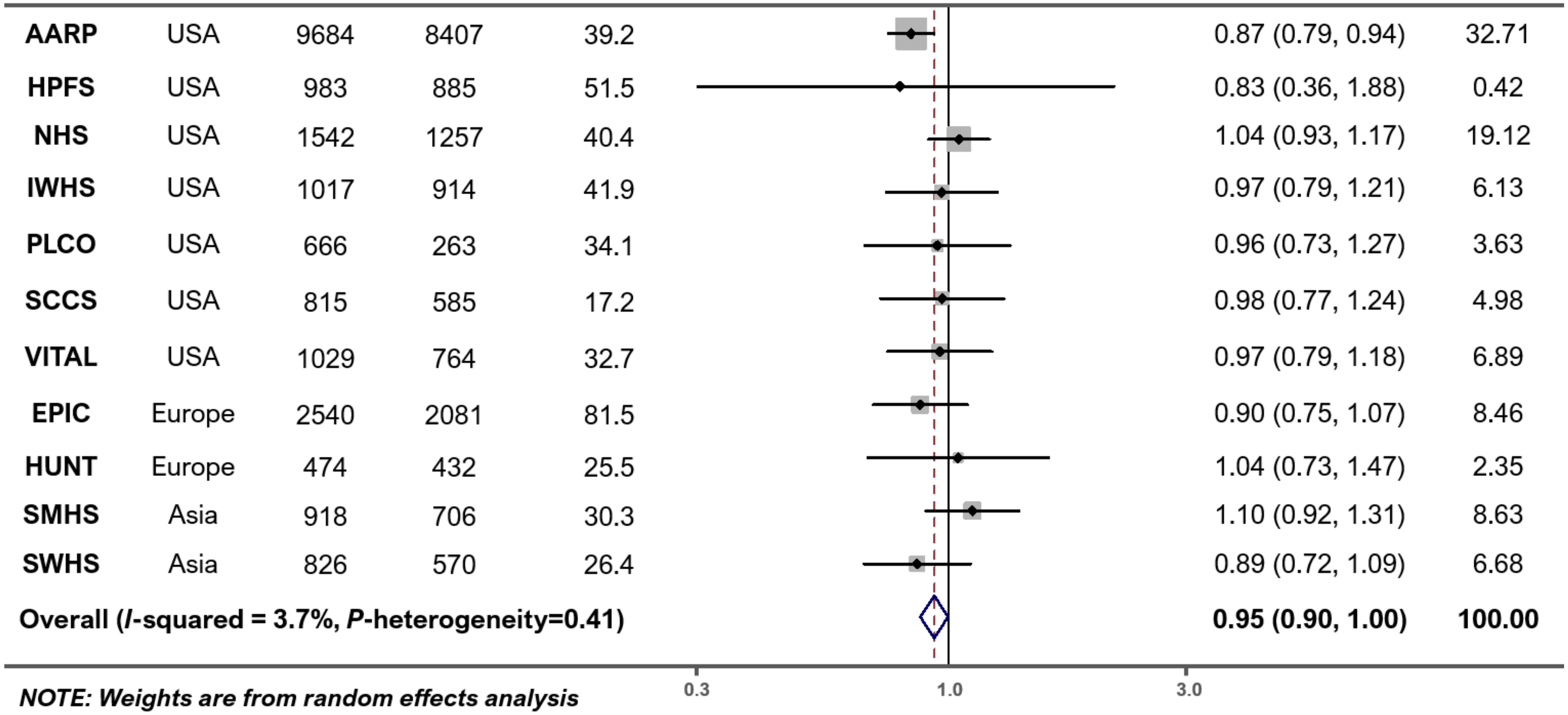

NOTE: Weights are from random effects analysis

Supplementary Figure 1. Cohort-specific associations of pre-diagnosis LTPA with all-cause mortality. Hazard ratios (95% CIs) for ≥8.3 MET-hours per week vs. none were shown after adjusting for age at diagnosis, sex, smoking status, smoking pack-years, race and ethnicity, education, alcohol consumption, history of diabetes, bod mass index levels, hormone therapy in women, histological type, tumor stage, and grade of lung cancer; and stratifying by cohort, year of lung cancer diagnosis, and time interval from leisure-time physical activity assessment to lung cancer diagnosis. Error bars represent 95% confidence intervals. All statistical tests were 2-sided. Abbreviations: AARP, National Health Institute-AARP Diet and Health Study; HPFS, Health Professionals Follow-up Study; NHS, Nurses' Health Study; IWHS, Iowa Women's Health Study; PLCO, Prostate, Lung, Colorectal and Ovarian Cancer Screening Trial; SCCS, Southern Community Cohort Study; VITAL, VITamins And Lifestyle Study; EPIC, European Prospective Investigation into Cancer & Nutrition; HUNT, Trøndelag Health Study; SMHS, Shanghai Men's Health Study; SWHS, Shanghai Women's Health Study; LTPA, leisure-time physical activity; MET-hr/wk, metabolic equivalent hours per week; HR, hazard ratio; CI, confidence interval;

Cohort	Country	No. Cases	No. Deaths	≥8.3 MET-hr/wk (%)	Adjusted HR (95% CI)	Weight (%)
**AARP**	USA	9372	6615	39.2	0.95 (0.87, 1.03)	23.42
**HPFS**	USA	983	812	51.5	1.35 (0.54, 3.34)	0.30
**NHS**	USA	1540	1171	40.4	1.02 (0.93, 1.12)	18.93
**IWHS**	USA	1016	741	41.9	1.06 (0.88, 1.28)	6.49
**PLCO**	USA	666	237	34.1	1.02 (0.81, 1.27)	4.67
**SCCS**	USA	711	481	17.2	0.93 (0.77, 1.11)	6.73
**VITAL**	USA	1020	658	32.8	1.03 (0.88, 1.22)	8.25
**EPIC**	Europe	2323	1666	82.3	0.91 (0.80, 1.04)	11.14
**SMHS**	Asia	918	684	30.3	1.16 (1.02, 1.32)	12.49
**SWHS**	Asia	826	531	26.4	0.96 (0.81, 1.14)	7.57
**Overall (*I*-squared = 18.2%, *P*-heterogeneity=0.28)**					**1.00 (0.95, 1.05)**	**100.00**

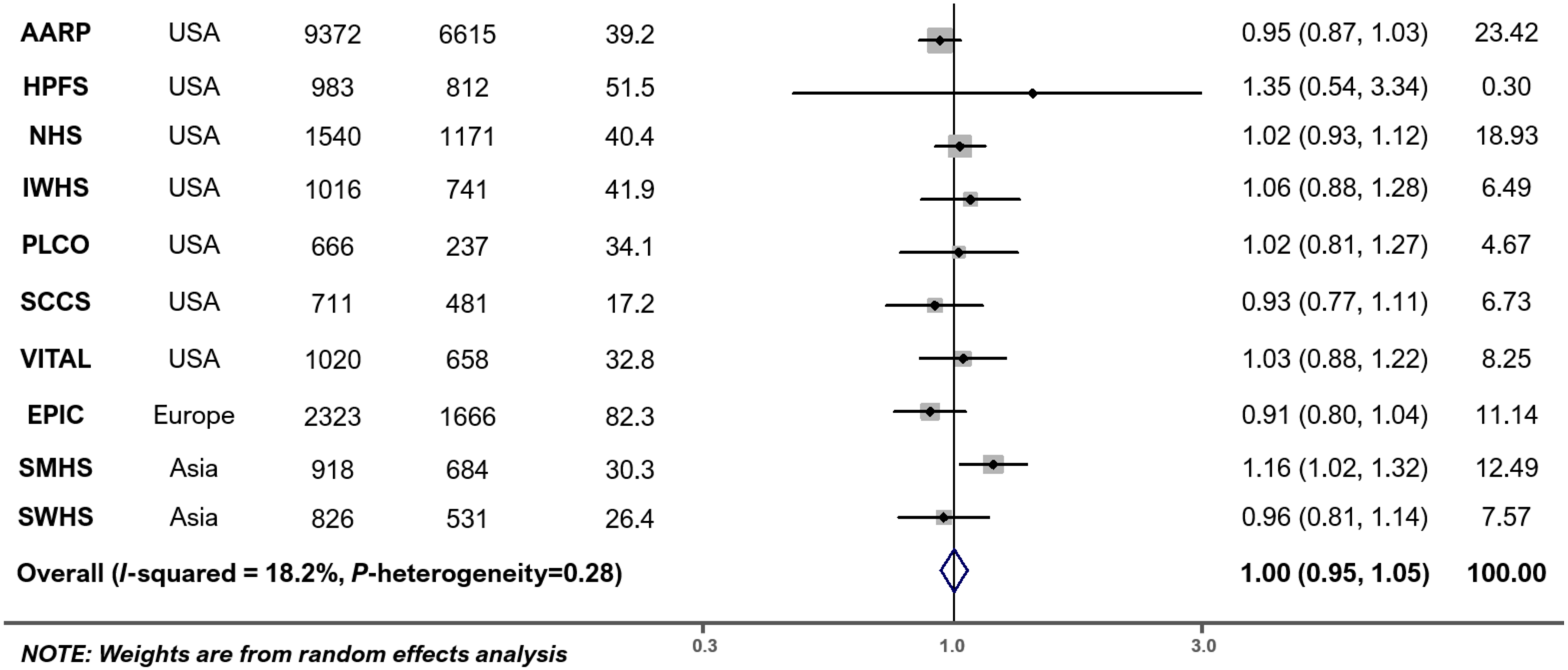

NOTE: Weights are from random effects analysis

Supplementary Figure 2. Cohort-specific associations of pre-diagnosis LTPA with lung cancer mortality. Cases who were missing cause of death were excluded from the analysis and death from other causes was treated as a competing risk. Trøndelag Health Study had no valid data on death causes. Hazard ratios (95% CIs) for ≥8.3 MET-hours per week vs. none were shown after adjusting for age at diagnosis, sex, smoking status, smoking pack-years, race and ethnicity, education, alcohol consumption, history of diabetes, body mass index levels, hormone therapy in women, histological type, tumor stage, and grade of lung cancer; and stratifying by cohort, year of lung cancer diagnosis, and time interval from leisure-time physical activity assessment to lung cancer diagnosis. Error bars represent 95% confidence intervals. All statistical tests were 2-sided. Abbreviations: AARP, National Health Institute-AARP Diet and Health Study; HPFS, Health Professionals Follow-up Study; NHS, Nurses' Health Study; IWHS, Iowa Women's Health Study; PLCO, Prostate, Lung, Colorectal and Ovarian Cancer Screening Trial; SCCS, Southern Community Cohort Study; VITAL, VITamins And Lifestyle Study; EPIC, European Prospective Investigation into Cancer & Nutrition; SMHS, Shanghai Men's Health Study; SWHS, Shanghai Women's Health Study; LTPA, leisure-time physical activity; MET-hr/wk, metabolic equivalent hours per week; HR, hazard ratio; CI, confidence interval;

Cohort	Country	No. Cases	No. Deaths	≥8.3 MET-hr/wk (%)	Adjusted HR (95% CI)	Weight (%)
**AARP**	USA	1364	920	41.4	0.79 (0.61, 1.03)	55.94
**IWHS**	USA	195	132	41.0	1.02 (0.56, 1.84)	11.19
**PLCO**	USA	319	46	33.2	0.59 (0.27, 1.30)	6.21
**SCCS**	USA	111	43	16.2	0.37 (0.08, 1.67)	1.69
**VITAL**	USA	205	83	29.8	0.51 (0.24, 1.09)	6.82
**EPIC**	Europe	259	147	81.8	0.85 (0.37, 1.97)	5.57
**SMHS**	Asia	158	66	26.6	1.48 (0.72, 3.05)	7.47
**SWHS**	Asia	165	45	23.0	0.70 (0.29, 1.68)	5.11
**Overall (*I*-squared = 0.0%, *P*-heterogeneity=0.48)**					**0.80 (0.66, 0.98)**	**100.00**

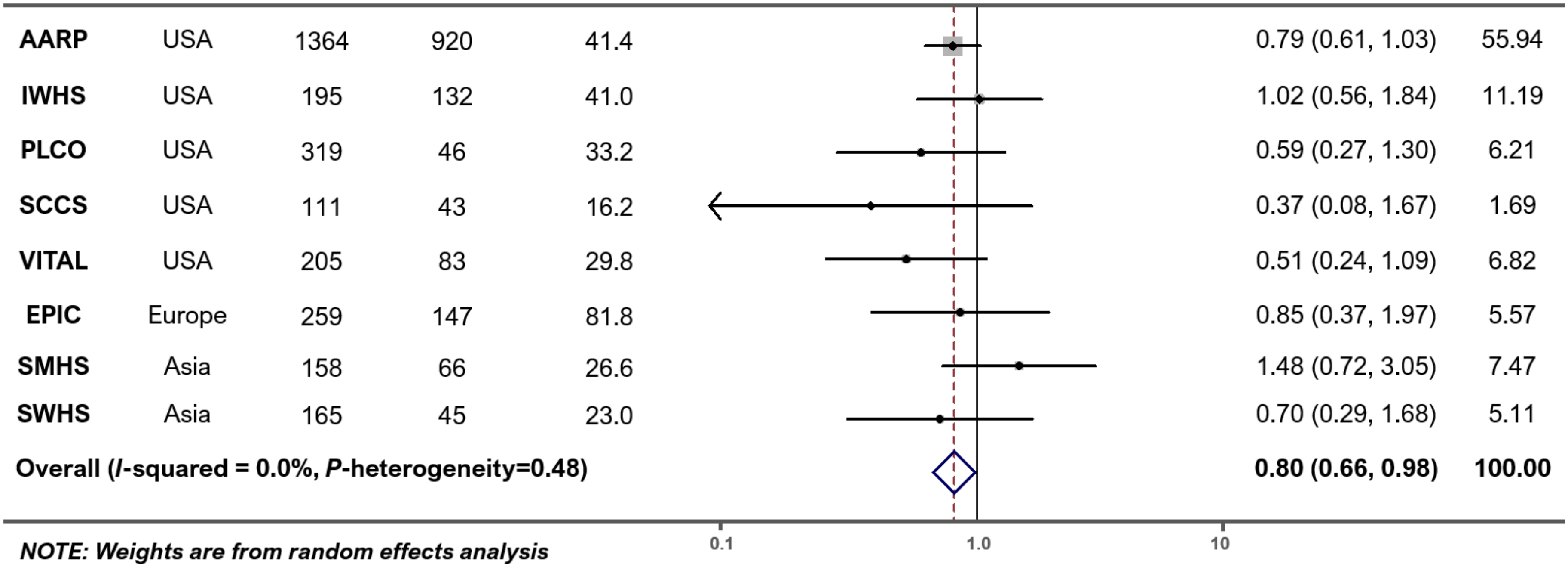

NOTE: Weights are from random effects analysis

Supplementary Figure 3. Cohort-specific associations of pre-diagnosis LTPA with all-cause mortality: Subgroup analysis among localized lung cancer cases. Cases who had missing on histologic type were excluded from the analysis. Health Professionals Follow-up Study, Nurses' Health Study, and Trøndelag Health Study had no valid data on lung cancer histologic type. Hazard ratios (95% CIs) for ≥8.3 MET-hours per week vs. none were shown after adjusting for age at diagnosis, sex, smoking status, smoking pack-years, race and ethnicity, education, alcohol consumption, history of diabetes, body mass index levels, hormone therapy in women, histological type, tumor stage, and grade of lung cancer; and stratifying by cohort, year of lung cancer diagnosis, and time interval from leisure-time physical activity assessment to lung cancer diagnosis. Error bars represent 95% confidence intervals. All statistical tests were 2-sided. Abbreviations: AARP, National Health Institute-AARP Diet and Health Study; IWHS, Iowa Women's Health Study; PLCO, Prostate, Lung, Colorectal and Ovarian Cancer Screening Trial; SCCS, Southern Community Cohort Study; VITAL, VITamins And Lifestyle Study; EPIC, European Prospective Investigation into Cancer & Nutrition; SMHS, Shanghai Men's Health Study; SWHS, Shanghai Women's Health Study; LTPA, leisure-time physical activity; MET-hr/wk, metabolic equivalent hours per week; HR, hazard ratio; CI, confidence interval;

Cohort	Country	No. Cases	No. Deaths	≥8.3 MET-hr/wk (%)	Adjusted HR (95% CI)	Weight (%)
**AARP**	USA	1335	546	41.5	0.72 (0.53, 0.98)	50.03
**IWHS**	USA	195	74	41.0	1.09 (0.52, 2.29)	8.64
**PLCO**	USA	319	33	33.2	0.69 (0.30, 1.60)	6.70
**SCCS**	USA	96	28	14.6	0.20 (0.01, 3.38)	0.59
**VITAL**	USA	205	52	29.8	0.42 (0.17, 1.04)	5.83
**EPIC**	Europe	249	124	81.1	0.66 (0.38, 1.16)	14.96
**SMHS**	Asia	158	63	26.6	1.30 (0.58, 2.94)	7.10
**SWHS**	Asia	165	41	23.0	1.15 (0.48, 2.77)	6.14
**Overall (*I*-squared = 0.0%, *P*-heterogeneity=0.50)**					**0.76 (0.61, 0.94)**	**100.00**

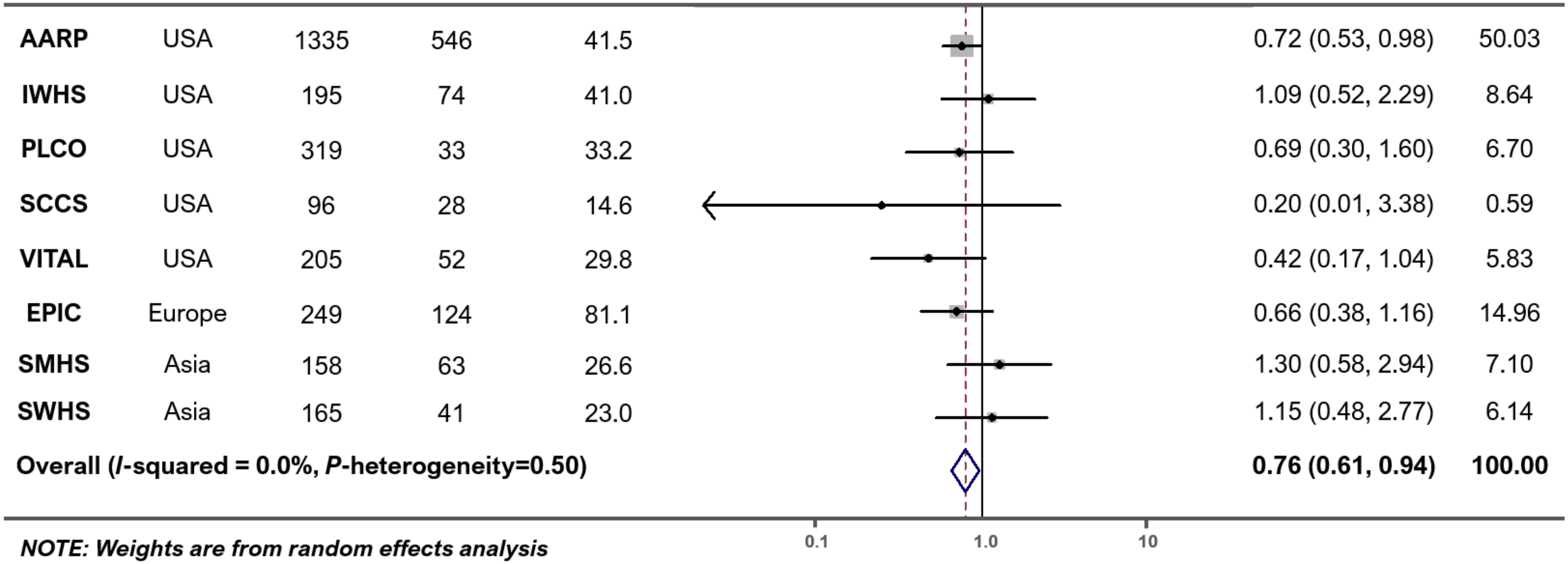

NOTE: Weights are from random effects analysis

Supplementary Figure 4. Cohort-specific associations of pre-diagnosis LTPA with lung cancer mortality: Subgroup analysis among localized lung cancer cases. Cases who were missing cause of death were excluded from the analysis and death from other causes was treated as a competing risk. Health Professionals Follow-up Study, Nurses' Health Study, and Trøndelag Health Study had no valid data on lung cancer histologic type. Trøndelag Health Study had no valid data on death causes. Hazard ratios (95% CIs) for ≥8.3 MET-hours per week vs. none were shown after adjusting for age at diagnosis, sex, smoking status, smoking pack-years, race and ethnicity, education, alcohol consumption, history of diabetes, body mass index levels, hormone therapy in women, histological type, tumor stage, and grade of lung cancer; and stratifying by cohort, year of lung cancer diagnosis, and time interval from leisure-time physical activity assessment to lung cancer diagnosis. Error bars represent 95% confidence intervals. All statistical tests were 2-sided. Abbreviations: AARP, National Health Institute-AARP Diet and Health Study; IWHS, Iowa Women's Health Study; PLCO, Prostate, Lung, Colorectal and Ovarian Cancer Screening Trial; SCCS, Southern Community Cohort Study; VITAL, VITamins And Lifestyle Study; EPIC, European Prospective Investigation into Cancer & Nutrition; SMHS, Shanghai Men's Health Study; SWHS, Shanghai Women's Health Study; LTPA, leisure-time physical activity; MET-hr/wk, metabolic equivalent hours per week; HR, hazard ratio; CI, confidence interval;
